# Supplementary material for: The Explosive Radiation of the Neotropical Tillandsia Subgenus Tillandsia (Bromeliaceae) Has Been Accompanied by Pervasive Hybridization
Source: Syst Biol. 2025 Jun 26;75(1):22–38. doi: 10.1093/sysbio/syaf039 (PMC12805668; doi:10.1093/sysbio/syaf039)
Supplement: syaf039_Supplemental_Files [file syaf039_supplemental_files.zip › supplemetary_captions.docx]

**Supporting files and tables**

**Supporting file 1 –** maximum-likelihood trees constructed for each of the 25 chromosomes. Eat tree was inferred on a dataset of concatenated SNPs with IQ-TREE, using substitution model TVMe+R2 with ascertainment bias correction. Branch lengths were calculated by number of substitutions per site and branch support was assessed using ultra-fast bootstrap estimation with 1,000 replicates.

**Supporting file 2** - Heatmaps summarizing 7,141 four-taxon D-statistic tests for each of the 25 reference chromosomes, indicated on each figure. *Tillandsia complanata* was used as the outgroup in all tests. The four taxa in each test have been rearranged to always obtain positive D values, and P2 and P3 are shown on the axes. Colour indicates the value of D and log value of p-value, as appears in legend (bottom right).

**Supporting file 3** - Examination of the effects of different filtering thresholds for minor alleles on different parts of the analysis. Using the explicit value of MAC (minor allele count) with values between 0-5, we performed maximum likelihood tree inference, species tree inference with quartet support and genome-wide D-statistic calculation (see main methods).

**Supporting Table S1.** sampled accessions in this study with collection, locality and voucher information. samples marked grey were removed from analysis (see table 3)

**Supporting Table S2.** List of species sampled for this study with clade assignment, phenotype and ecology (if known). Sheet 2 details references to corresponding literature.

**Supporting Table S3.** Samples accessions with information about sequence read number, alignment rates and number of sequences after filtering. samples marked grey were removed from analysis.

**Supporting Table S4.** Description of data-sets used in main analysis in the study, their properties and use in analysis.
