# Supplementary material for: The Explosive Radiation of the Neotropical Tillandsia Subgenus Tillandsia (Bromeliaceae) Has Been Accompanied by Pervasive Hybridization
Source: Syst Biol. 2025 Jun 26;75(1):22–38. doi: 10.1093/sysbio/syaf039 (PMC12805668; doi:10.1093/sysbio/syaf039)

Supporting Figures

Supporting Figure S1..2
Supporting Figure S2..3
Supporting Figure S3..4
Supporting Figure S4..6
Supporting Figure S5..7
Supporting Figure S6..8
Supporting Figure S7..9
Supporting Figure S8..10

Supporting Figure S1. Maximum-likelihood (ML) phylogenomic tree inferred on a supermatrix partitioned by genes with IQ-TREE2, using ModelFinder to select the best-fitting partitioning scheme and models. Branch lengths were calculated by number of substitutions per site and branch support was assessed using ultra-fast bootstrap estimation with 1,000 replicates. Colors follow the clades in Figure 2.

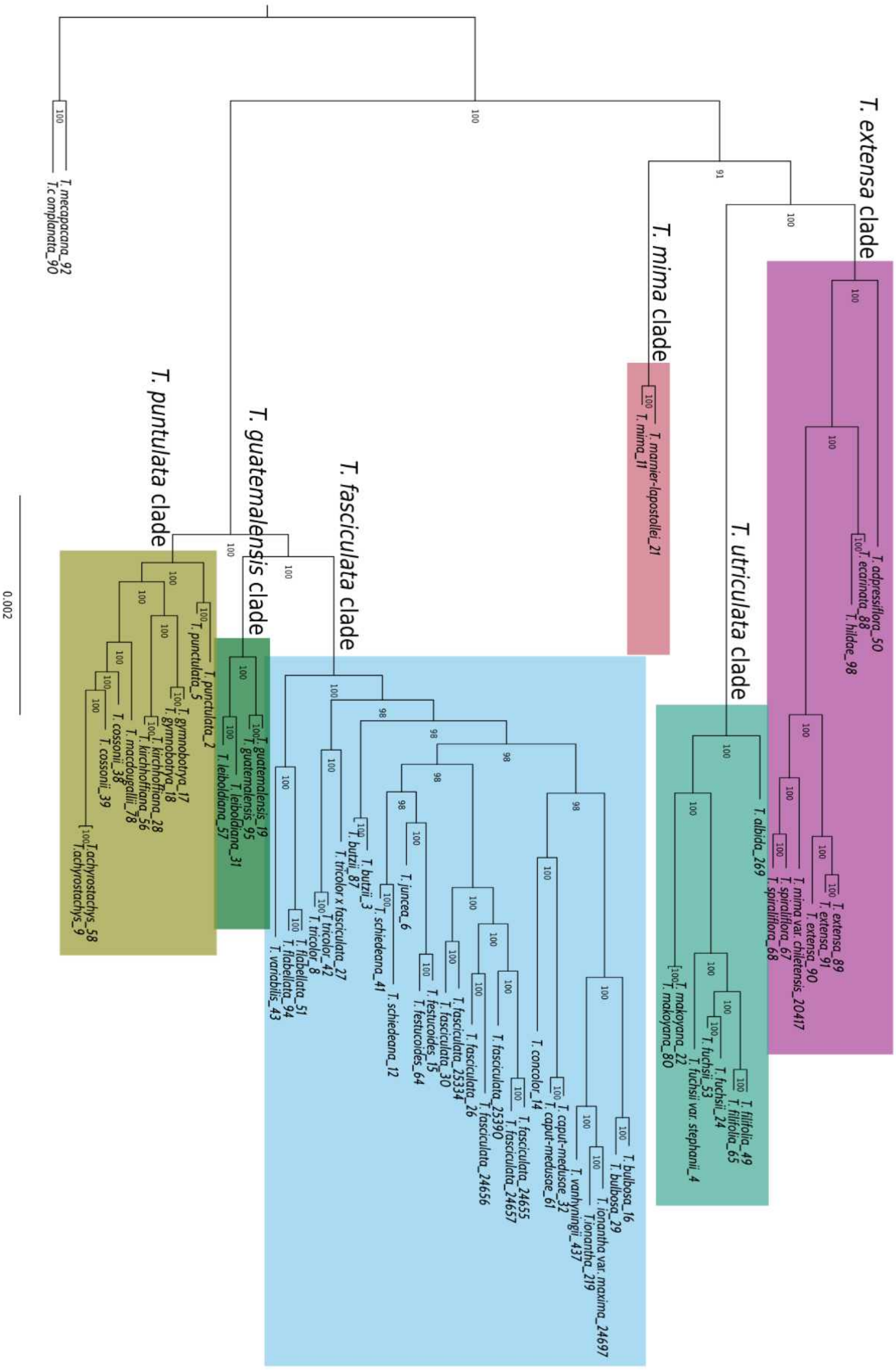

Supporting Figure S2. *Tillandsia zoquensis* is monophyletic with *T. fasciculata*. A maximum-likelihood tree was inferred on a dataset of concatenated SNPs with IQ-TREE, using substitution model TVMe+R2 with ascertainment bias correction. For sample collection details, see Supporting Table S2.

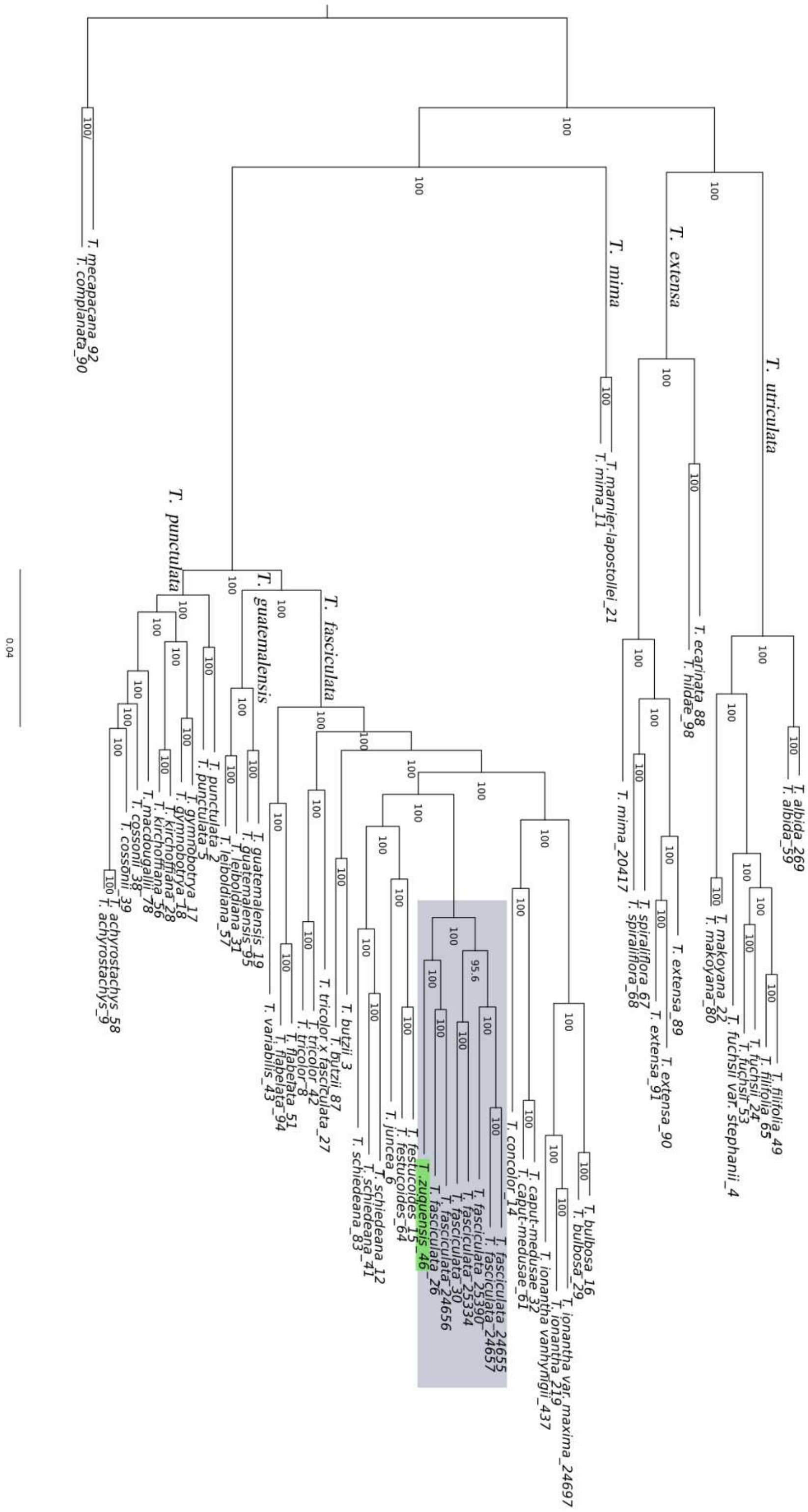

Supporting Figure S3. Visualisation of gene tree discordance within the data-set. Coalescent-based species tree was generated on 15,791 genomic windows using ASTRAL-III. For each branch, the top number indicates the number of gene trees concordant with the species tree at that node and the bottom number indicates the number of gene trees in conflict with that species tree. Pie charts at the nodes show levels of gene tree discordance: the percentages of concordant gene trees (blue), the top alternative bipartition (green), other conflicting topologies (red) and uninformative gene trees (grey).

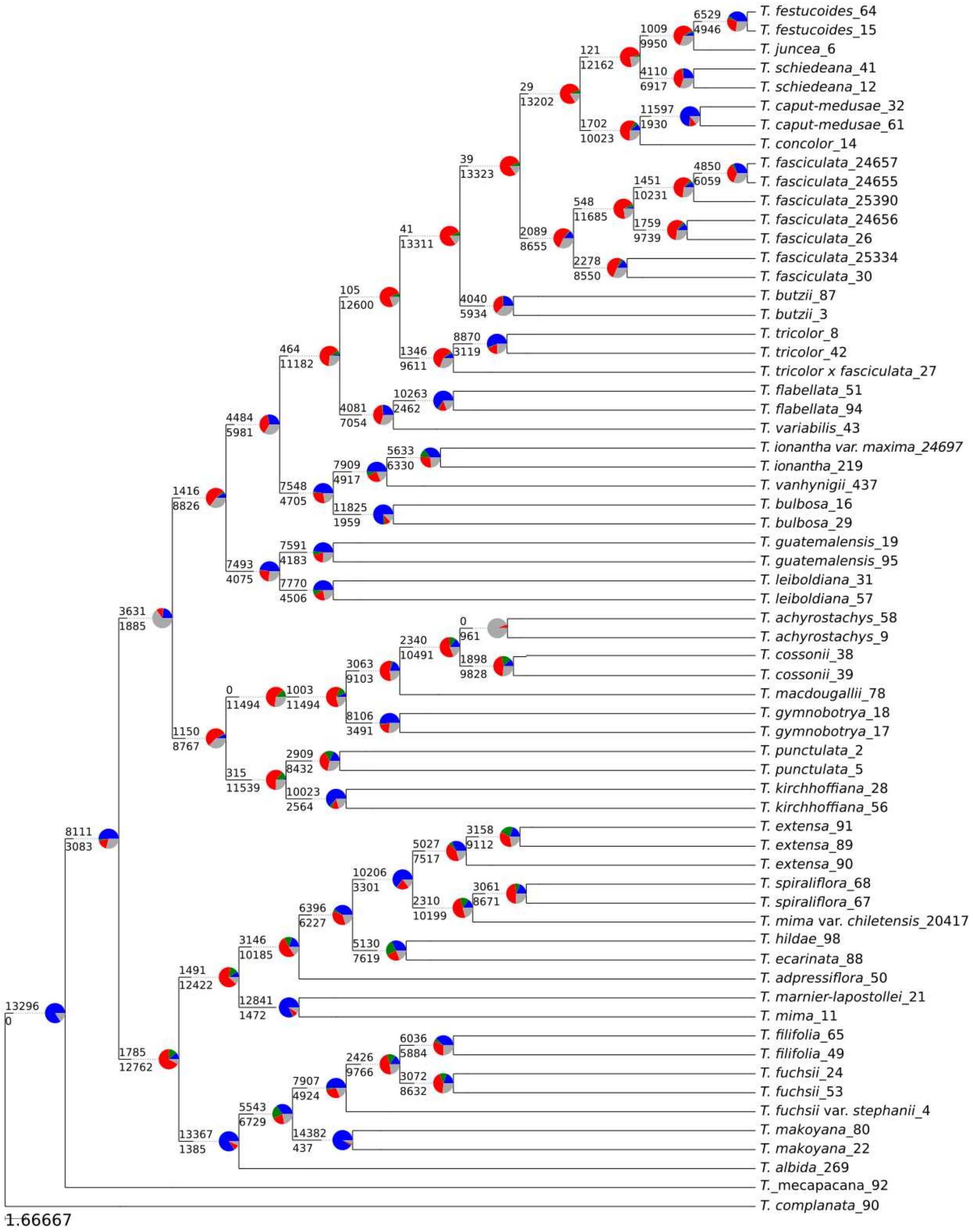

6529
4946
T. festucoides_64
T. festucoides_15
1009
9950
T. juncea_6
121
12162
4110
6917
T. schiedeana_41
T. schiedeana_12
29
13202
11597
1930
T. caput-medusae_32
T. caput-medusae_61
1702
10023
T. concolor_14
39
13323
4850
6059
T. fasciculata_24657
T. fasciculata_24655
1451
10231
T. fasciculata_25390
548
11685
1759
9739
T. fasciculata_24656
T. fasciculata_26
41
13311
2089
8655
2278
8550
T. fasciculata_25334
T. fasciculata_30
105
12600
4040
5934
T. butzii_87
T. butzii_3
8870
3119
T. tricolor_8
T. tricolor_42
464
11182
1346
9611
T. tricolor x fasciculata_27
10263
2462
T. flabellata_51
T. flabellata_94
4484
5981
4081
7054
T. variabilis_43
5633
6330
T. ionantha var. maxima_24697
T. ionantha_219
7909
4917
T. vanhynigii_437
1416
8826
7548
4705
11825
1959
T. bulbosa_16
T. bulbosa_29
7591
4183
T. guatemalensis_19
T. guatemalensis_95
7493
4075
7770
4506
T. leiboldiana_31
T. leiboldiana_57
3631
1885
0
961
T. achyrostachys_58
T. achyrostachys_9
2340
10491
1898
9828
T. cossonii_38
T. cossonii_39
3063
9103
0
11494
1003
11494
T. macdougallii_78
8106
3491
T. gymnobotrya_18
T. gymnobotrya_17
1150
8767
2909
8432
T. punctulata_2
T. punctulata_5
315
11539
10023
2564
T. kirchhoffiana_28
T. kirchhoffiana_56
8111
3083
3158
9112
T. extensa_91
T. extensa_89
5027
7517
T. extensa_90
10206
3301
3061
8671
T. spiraliflora_68
T. spiraliflora_67
6396
6227
2310
10199
T. mima var. chiletensis_20417
5130
7619
T. hildae_98
T. ecarinata_88
3146
10185
T. adpressiflora_50
1491
12422
12841
1472
T. marnier-lapostollei_21
T. mima_11
13296
0
6036
5884
T. filifolia_65
T. filifolia_49
2426
9766
3072
8632
T. fuchsii_24
T. fuchsii_53
7907
4924
T. fuchsii var. stephanii_4
1785
12762
5543
6729
14382
437
T. makoyana_80
T. makoyana_22
13367
1385
T. albida_269
T._mecapacana_92
T. complanata_90
1.66667

Supporting Figure S4. Widespread deviations from tree-like structure in *Tillandsia* subgenus *Tillandsia* phylogeny. A Cladogram of 100 randomly selected ML phylogenetic trees inferred from non-overlapping 10kb genomic windows. Branch lengths were forced into ultrametric structure for visualisation purposes only. Colours follow the legend and Figure 2.

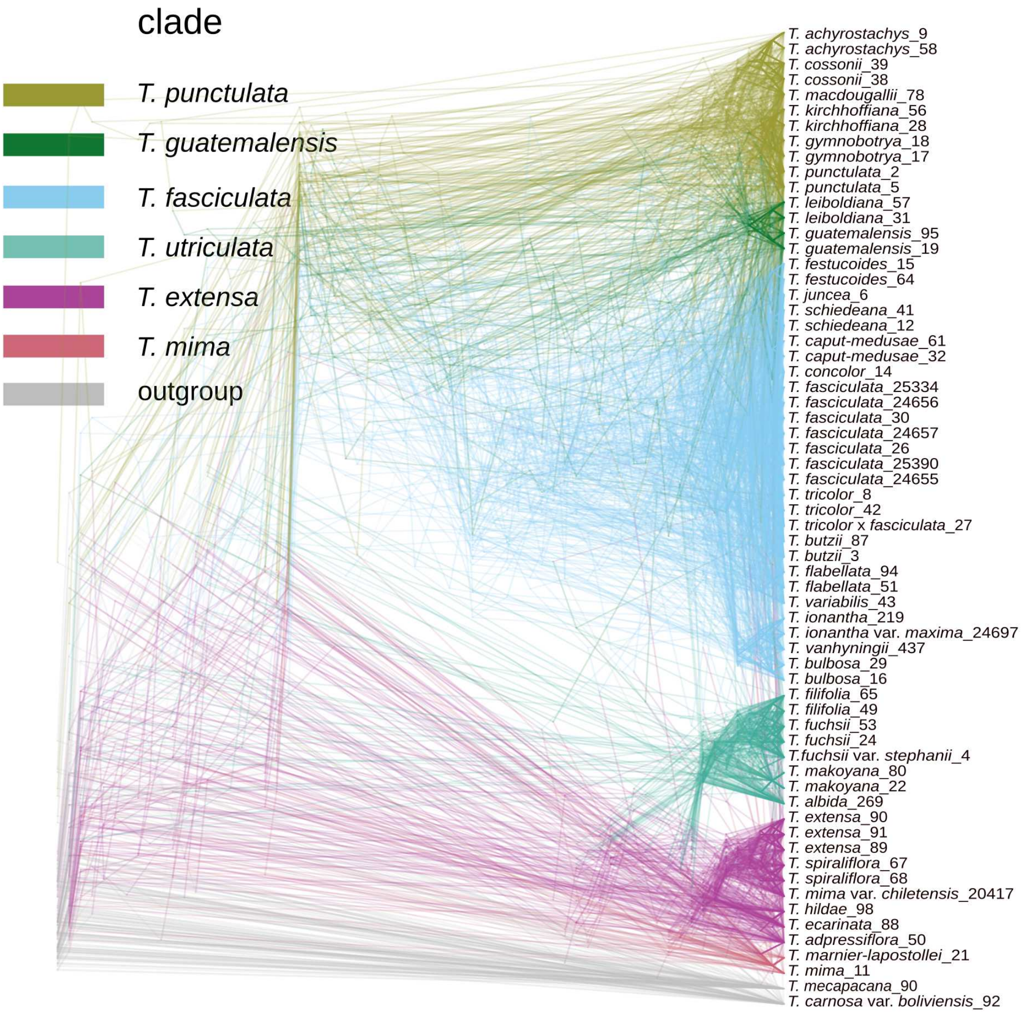

Supporting Figure S5. a, Derived alleles conveying deviations from tree structure in the Central American K subclades of *Tillandsia* subgenus *Tillandsia*. The Euler diagram represents the proportion of significantly elevated D_{min} scores for comparisons in trios including species from within or between clades. Numbers indicate the total number of significantly elevated D_{min} for each overlap. **b**, PCA analysis for all clades on bi-allelic, distance-pruned 16,204 SNPs, allowing maximum 10% missing data.

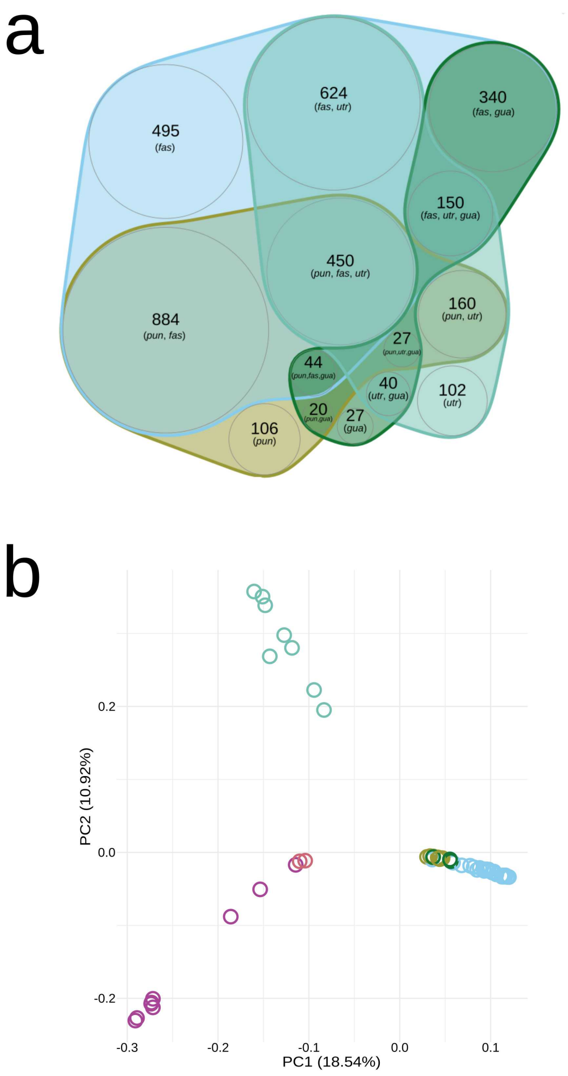

Supporting Figure S6. Variation along the genome in relationships between the major *Tillandsia subgenus Tillandsia* subclades. Visualisation was produced using topology weighting by iterative sampling of subtrees in genomic windows of 50 SNPs. Colours represent the frequency of each topology along chromosomal position in each chromosome: white sectors indicate regions excluded due to high levels of missing data, including highly repetitive regions. Two chromosomes are presented: chromosome 4, to depict typical distribution of the main topologies, and chromosome 18, to contrast.

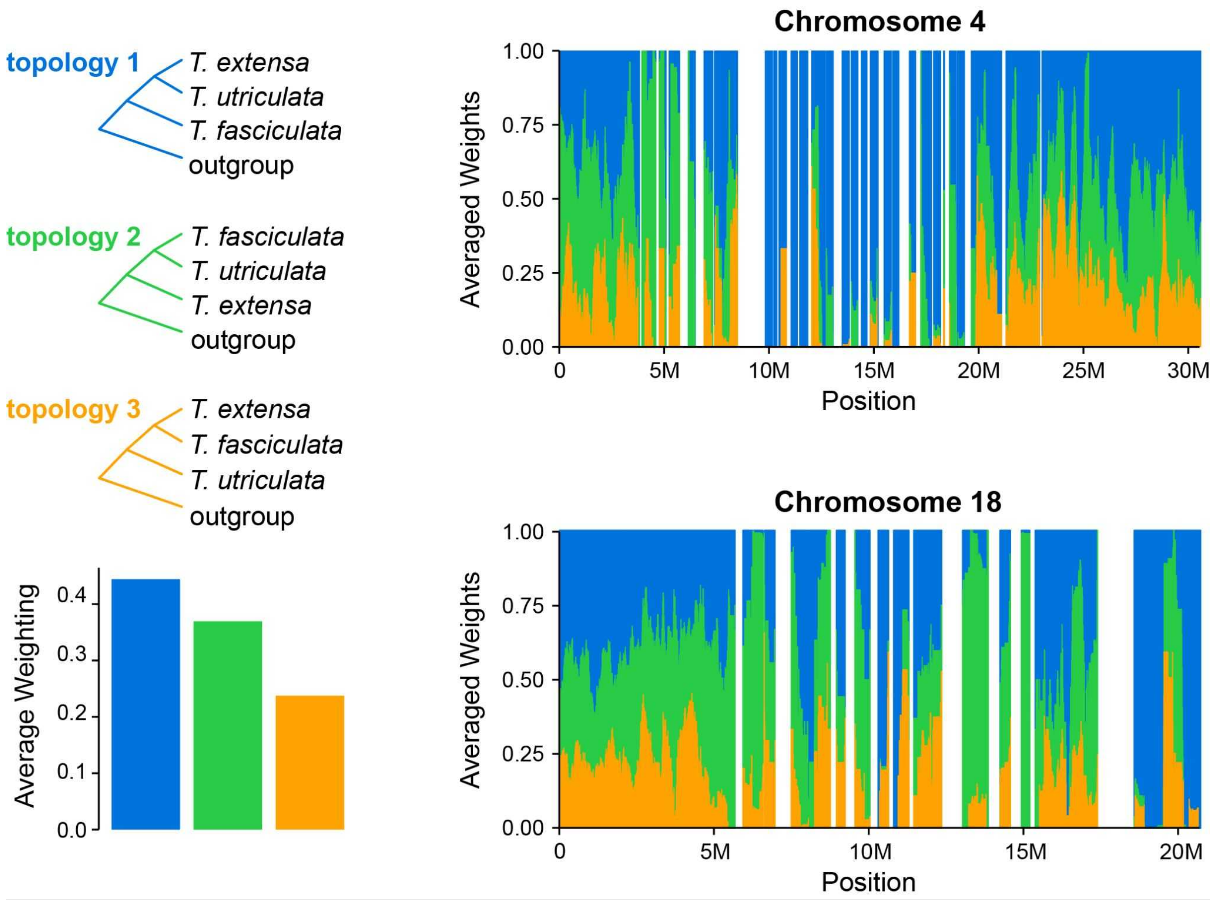

Supporting Figure S7. Heatmap summarizing 7,141 four-taxon D-statistic tests. *Tillandsia complanata* was used as the outgroup in all tests. The four taxa in each test have been rearranged to always obtain positive D values and P2 and P3 are shown on the axes. Colour indicates the value of D and the log value of p-value, the latter estimated using a block jackknife procedure with a 200 kb window size and corrected for family-wise error rate. Colours on the bars correspond to the subclades in Figure 2.

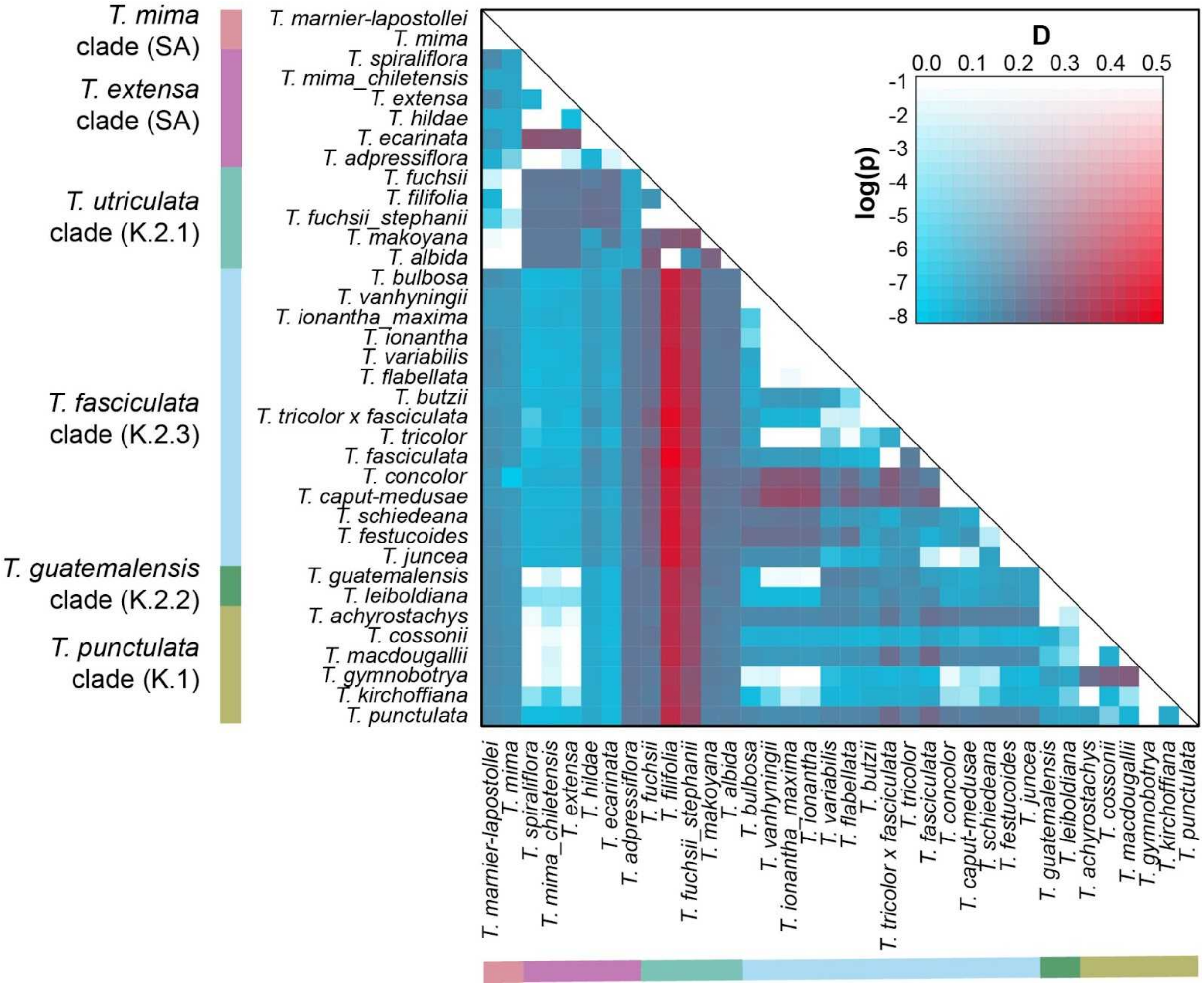

Supporting Figure S8. Heatmap summarising 7,141 $f4$-ratio tests. *Tillandsia complanata* was used as the outgroup for all tests. Color indicates the value of $f4$ and log value of p-value, the latter estimated using a block jackknife procedure with a 200 kb window size and corrected for family wise error rate. Colors correspond to the clades in Figure 2.

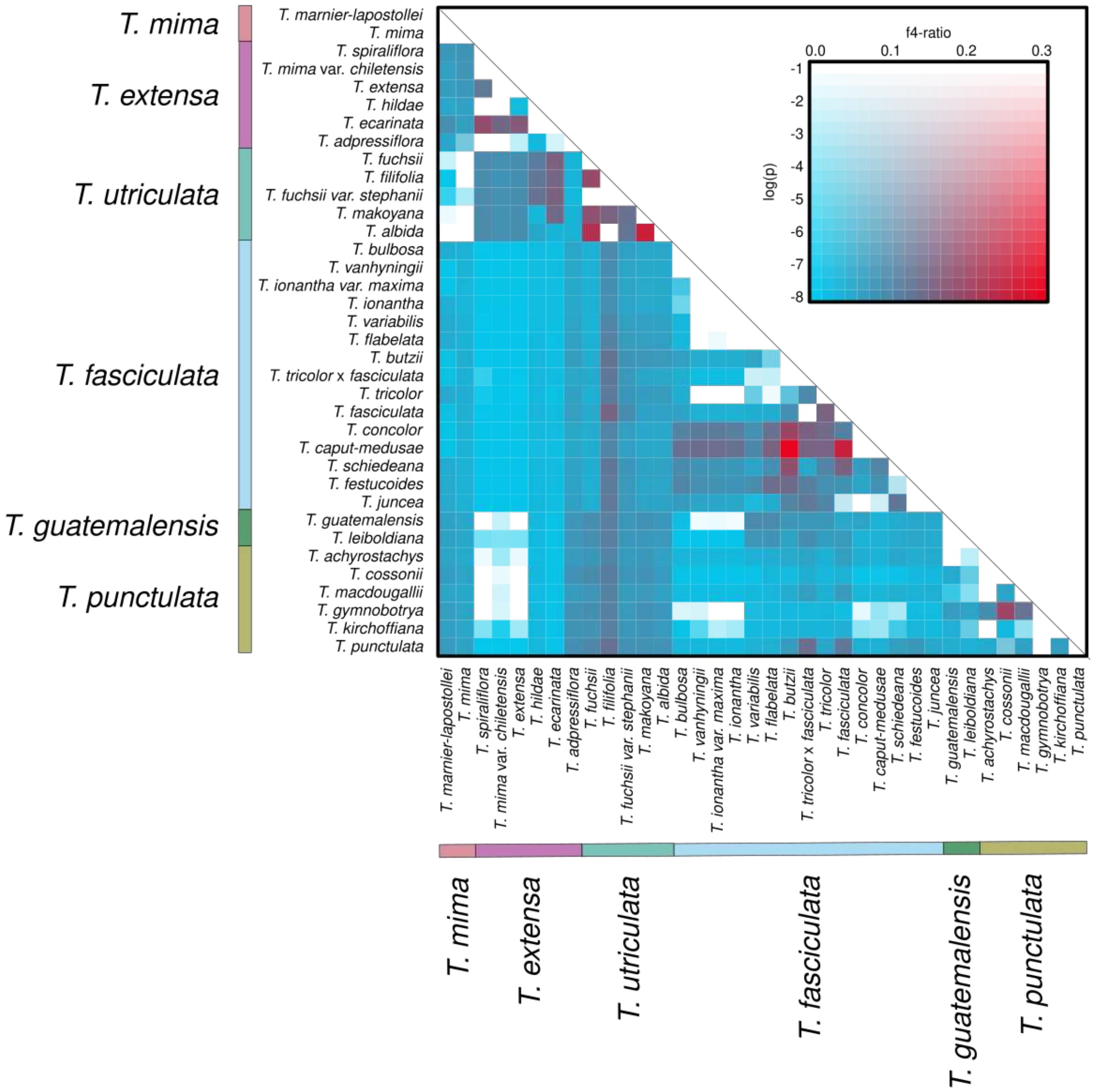

Supplement: syaf039_Supplemental_Files [file syaf039_supplemental_files.zip › Yardeni_et_al._SysBio_supporting_figures.pdf]
